# Mechanisms for Development of Ciprofloxacin Resistance in a Clinical Isolate of *Pseudomonas aeruginosa*

**DOI:** 10.3389/fmicb.2020.598291

**Published:** 2021-01-08

**Authors:** Congjuan Xu, Huimin Liu, Xiaolei Pan, Zhenzhen Ma, Dan Wang, Xinxin Zhang, Guangbo Zhu, Fang Bai, Zhihui Cheng, Weihui Wu, Yongxin Jin

**Affiliations:** ^1^State Key Laboratory of Medicinal Chemical Biology, Key Laboratory of Molecular Microbiology and Technology of the Ministry of Education, Department of Microbiology, College of Life Sciences, Nankai University, Tianjin, China; ^2^Tianjin Union Medical Center, Nankai University Affiliated Hospital, Tianjin, China

**Keywords:** *Pseudomonas aeruginosa*, ciprofloxacin resistance, *mexS*, *gyrA*, MexEF-OprN

## Abstract

Treatment of infections by *Pseudomonas aeruginosa* is difficult due to its high intrinsic and acquired antibiotic resistance. Upon colonization in the human hosts, *P. aeruginosa* accumulates genetic mutations that confer the bacterium antibiotic resistance and ability to better live in the host environment. Characterizing the evolutionary traits would provide important insights into the development of effective combinatory antibiotic therapies to cure *P. aeruginosa* infections. In this work, we performed a detailed analysis of the molecular mechanisms by which a clinical isolate (CSP18) yields a ciprofloxacin-resistant derivative (CRP42). Genomic DNA re-sequencing and RNAseq were carried out to compare the genomic mutational signature and transcriptional profiles between the two isolates. The results indicated that D87G mutation in GyrA, together with MexEF-OprN hyper-expression caused by F7S mutation in MexS, was responsible for the increased resistance to ciprofloxacin in the isolate CRP42. Further simulation of CRP42 by gene editing in CSP18 demonstrated that D87G mutation in GyrA rendered CSP18 a fourfold increase in minimum inhibitory concentration against ciprofloxacin, while F7S mutation in MexS conferred an additional eightfold increase. Our experimental results demonstrate for the first time that the clinically relevant F7S point mutation in MexS results in hyper-expression of the *mexEF-oprN* and thus confers *P. aeruginosa* resistance to ciprofloxacin.

## Introduction

*Pseudomonas aeruginosa* is an opportunistic human pathogen and one of the leading causes of nosocomial infections all over the world (Vincent et al., 1995). Infections by *P. aeruginosa* are often challenging to cure due to its intrinsic and acquired resistance to a wide variety of antibiotics, leaving a limited number of effective antimicrobial agents. Ciprofloxacin is one of the most important antibiotics used for the treatment of *P. aeruginosa* infections (Andriole, 2005), including children and adults with cystic fibrosis (Doring et al., 2000). However, ciprofloxacin resistance of clinical *P. aeruginosa* isolates has been increasingly reported worldwide (Pitt et al., 2003; Landman et al., 2007). For example, 30% of clinical *P. aeruginosa* isolated from cystic fibrosis patients were ciprofloxacin resistant in a study from the United Kingdom (Pitt et al., 2003), while in a study from New York, it was 40% (Landman et al., 2007).

The mechanisms of ciprofloxacin resistance in *P. aeruginosa* are usually multifactorial, which include the following: (i) mutation of ciprofloxacin target-encoding genes *gyrAB* (encoding DNA gyrase) or/and *parCE* (encoding topoisomerase; Lee et al., 2005; Feng et al., 2019); (ii) overexpression of efflux pump encoding genes to increase the expulsion of ciprofloxacin from *P. aeruginosa* cells, including *mexEF-oprN*, *mexAB-oprM*, *mexXY*, and *mexCD-oprJ* (Kohler et al., 1997; Masuda et al., 2000; Rehman et al., 2019); and (iii) acquisition of ciprofloxacin-resistant genes through horizontal gene transfer (Chavez-Jacobo et al., 2018; Liu et al., 2018). Although these and other studies have associated the mechanisms of ciprofloxacin resistance among clinical *P. aeruginosa* strains, there is little information about the molecular details resulting in the evolutionary dynamics of clinical isolates of *P. aeruginosa* from ciprofloxacin susceptible to resistant, as well as the relative quantitative contribution of each of the resistant mechanisms mentioned above.

In this work, two clinical strains of *P. aeruginosa* were obtained from sputum samples of the same patient with ulcerative colitis before and after treatment first with cefoxitin for 7 days and then imipenem-cilastatin sodium and ciprofloxacin for another 8 days. The first strain was isolated soon after the patient was admitted to the hospital, while the second one was obtained 15 days after the antibiotics treatment. The first isolate, CSP18, was ciprofloxacin susceptible, whereas the latter one, CRP42, was ciprofloxacin resistant. Therefore, our aim was to characterize the molecular mechanisms for the ciprofloxacin resistance being developed in the clinical setting. The results presented in this study demonstrated that a D87G mutation in GyrA combined with hyper-expression of *mexEF-oprN* caused by an F7S mutation in MexS are the contributory factors for the conversion. Furthermore, we present experimental evidence that D87G mutation of GyrA contributes a fourfold increase in minimum inhibitory concentration (MIC) to ciprofloxacin, while MexEF-OprN hyper-expression caused by the F7S mutation in MexS contributes a further eightfold increase of MIC to ciprofloxacin in the CRP42 strain. These findings provide novel insights into the *mexEF-oprN* overexpression and ciprofloxacin resistance in *P. aeruginosa*.

## Materials and Methods

### Basic Characterization of the *P. aeruginosa* Isolates

Bacterial strains and plasmids used in this work are shown in Supplementary Table 1. Ciprofloxacin-susceptible (CSP18) and -resistant (CRP42) *P. aeruginosa* strains characterized here were isolated from sputum samples of a patient with ulcerative colitis before and after treatment with antibiotics for 15 days at the Nankai University Affiliated Hospital, Tianjin, China. The 16S rDNA was PCR amplified with primers (Supplementary Table 2) and sequenced to identify the species of the two isolates (Spilker et al., 2004). RAPD (Random amplified polymorphic DNA) typing was performed with primer 272 following a previous description (Mahenthiralingam et al., 1996). Multilocus sequence typing (MLST) was carried out as described previously with minor modification to confirm the allelic profiles of these two isolates (Curran et al., 2004). Briefly, chromosomal DNA, purified from overnight cultured bacteria with DNA purification kit (Tiangen Biotec, Beijing, China), was used as PCR template. The internal fragments of *aroE*, *acsA*, *mutL*, *guaA*, *ppsA*, *nuoD*, and *trpE* genes were PCR-amplified, sequenced using primers (Supplementary Table 2) as described previously and then the sequences were submitted to the *P. aeruginosa* MLST database^1^ to obtain the allelic numbers. A sequence type (ST) was assigned to each *P. aeruginosa* isolate by combination of the seven allelic numbers. MIC of antibiotics was examined by the twofold serial dilution method described previously except for the bacteria being grown in LB broth (Richardot et al., 2016), and susceptibility was interpreted based on the Clinical and Laboratory Standards Institute guidelines (CLSI 2011–2018).

### Plasmids Construction and Gene Editing

For expression of *gyrA*, a 3338-bp *gyrA*-containing fragment with its putative promoter and SD (Shine-Dalgarno) sequence was amplified with CSP18 and CRP42 chromosomal DNA as templates (primers listed in Supplementary Table 2). The PCR products were treated with *Sac*I and *Hin*dIII and then cloned into vector pUC18T-mini-Tn7T, leading to pUC18T-*gyrA*_CSP__18_ and pUC18T-*gyrA*_CRP__42_, respectively.

To delete the *gyrA* gene, a 924-bp fragment immediately upstream of the *gyrA* gene and an 836-bp fragment downstream of the *gyrA* were amplified (primers listed in Supplementary Table 2). The two fragments were digested with *Eco*RI–*Kpn*I and *Kpn*I–*Bam*HI, respectively, and then ligated into pEX18Tc that was treated with *Eco*RI–*Bam*HI, leading to pEX18-*gyrA*. To make the *mexS* (MexS-F7S) point mutation construct, F7S point mutated sites were included in the reverse primer of upstream and forward primer of downstream of *mexS* (primers shown in Supplementary Table 2), and the upstream and downstream fragments were digested with *Eco*RI–*Bgl*II and *Bgl*II–*Hin*dIII, and then ligated into pEX18Tc vector, leading to pEX18-*mexS*. Gene editing in *P. aeruginosa* was performed by conjugal transfer of these constructs and then selecting for single and double crossovers, as described previously (Schweizer, 1992).

### RNA Extraction, RT-qPCR, and RNAseq Analysis

Cultured *P. aeruginosa* strains were subcultured to an optical density of 1.0 (wavelength of 600 nm). RNA was purified with an RNAprep Pure Cell/Bacteria Kit (Tiangen Biotec, Beijing, China) and then reverse transcribed to cDNA with PrimeScript Reverse Transcriptase and random primers (Takara). The cDNA was added into the mixture of indicated qRT-PCR primers (shown in Supplementary Table 2) and SYBR premix Ex Taq II (Takara) and then quantitatively PCR amplified with a CFX Connect Real-time system (Bio-Rad, United States). A 30S ribosomal protein gene *rpsL* was utilized as an internal control.

The RNAseq analysis was carried out by GENEWIZ (Suzhou, China) as previously described (Xu et al., 2020). Briefly, total RNA was isolated from *P. aeruginosa* strains as described above, qualified and quantified by Agilent 2100 Bioanalyzer (Agilent Technologies, Palo Alto, CA, United States), NanoDrop, and electrophoresis on 1% (wt/vol) agarose gel. After removing the rRNA by Ribo-Zero rRNA Removal Kit (Illumina for bacteria), the mRNA was fragmented and reverse transcribed. Then, the purified double-strand cDNA was subjected to end repair and ligation with adaptors. Following 11 cycles of amplification with PCR, the products were cleaned, validated using an Agilent 2100 Bioanalyzer (Agilent Technologies, Palo Alto, CA, United States), and further quantified with a Qubit 2.0 Fluorometer (Invitrogen, Carlsbad, CA, United States). The generating libraries were sequenced on an Illumina HiSeq 2500 platform with a 2 × 150 paired-end (PE) configuration.

Sequence reads were aligned to PAO1 reference genome (NC_002516.2) using software Bowtie2 (v2.1.0). RNA expressional levels were analyzed by HTSeq (v0.6.1p1). Genes with differential expression were identified using the DESeq Bioconductor package, with the fold change larger than 2 and *P* value no more than 0.05 as cutoff values.

### DNA Isolation and Genomic Re-sequencing

Genomic DNA isolation and re-sequencing were carried out as described previously (Xu et al., 2020). Briefly, genomic DNA of *P. aeruginosa* strains was purified with DNA purification kit (Tiangen Biotec, Beijing, China). Two hundred nanograms of genomic DNA was used to generate fragments smaller than 500 bp with sonication (Covaris S220). After end treatment and adaptor ligation, the DNA fragments of about 470 bp were purified and amplified with PCR for six cycles. The PCR products were then cleaned up using beads, validated, and quantified using Qubit3.0 Fluorometer (Invitrogen, Carlsbad, United States). Sequencing was performed on an Illumina Hiseq instrument with a 2 × 150 PE configuration following the manufacturer’s instructions (Illumina, San Diego, United States). The sequences were mapped onto the PAO1 reference genome (NC_002516.2) with software BW2 (version 0.7.12). InDel mutation and single nucleotide variation (SNV) were analyzed via the Unified Genotyper module from GATK and the software Samtools (version 1.1).

### PCR and Sequencing of *mexS, mexT* and *gyrA*

The full-length *mexS* gene with its 85-bp upstream and 87-bp downstream region was PCR amplified using primers displayed in Supplementary Table 2, cloned into pUCP20. Similarly, the *mexT* gene with its 114-bp upstream and 77-bp downstream region was PCR amplified and cloned into pUCP20. After sequencing, the *mexS* or *mexT* gene sequence from CSP18 or CRP42 was aligned with the *mexS* or *mexT* gene of the PAO1 and PA14 reference strains^2^. For *gyrA*, the *gyrA* gene with its 467-bp upstream and 99-bp downstream region was PCR amplified, cloned into pUC18T-mini-Tn7T, and analyzed by sequencing. The *gryA* gene sequence from CSP18 or CRP42 was aligned with the *gryA* gene of the PAO1 reference strain (see text footnote 2).

### Quantification of Pyocyanin

The pyocyanin production of *P. aeruginosa* was determined as previously described with minor modification (Tan et al., 2016). Briefly, overnight bacterial culture was inoculated with 50-fold dilution and grown for 24 h. One-milliliter supernatant of each bacterial culture was extracted into 0.5 ml of chloroform, 0.4 ml of which was then re-extracted into 0.3 ml of HCl (0.2 N), and absorbance at 520 nm was then determined with Varioskan Flash (Thermo Scientific, Netherlands).

### Rhamnolipid Production Assay

Rhamnolipid production was observed by growing bacteria on an M8-based agar plate as previously described (Chen et al., 2019). Bacteria were cultured in LB medium overnight and 1 μl of the culture was inoculated onto the plate and grown for 24 h at 37°C. After that, the plate was further kept at room temperature for more than 72 h until a blue halo appeared surrounding the colony. The relative production level of rhamnolipids was examined by measuring the diameter of each halo divided by the diameter of its colony.

### Statistical Analysis

Statistical analyses were performed with GraphPad software. The real-time qPCR results were analyzed by Student’s *t* test (two-tailed).

### Ethics Statement

We have a waiver from the medical ethics committee of Tianjin Union Medical Center, exempting this work from the requirement for ethics approval and written informed consent as the clinical strains used in the study come from the routine procedures of the clinical laboratory rather than the clinical trials.

### Data Availability Statement

The datasets generated for the present work can be found in NCBI, under accession PRJNA638759.

## Results

### Clinical Isolates CSP18 and CRP42 Belong to the Same Clone Lineage

Sputum samples from a patient with ulcerative colitis were collected before and after treatment first with cefoxitin for 7 days and then imipenem-cilastatin sodium and ciprofloxacin for another 8 days. Two isolates were obtained, one before the antibiotics treatment that was sensitive to ciprofloxacin (CSP18), and the other one after the antibiotics treatment that displayed resistance against the ciprofloxacin (CRP42; Table 1). PCR amplification and sequencing analysis of the 16S rDNAs demonstrated that they were both *P. aeruginosa* (Supplementary Figure 1A). The MLST analysis for both strains revealed an allelic profile for *aroE*, *acsA*, *mutL*, *guaA*, *ppsA*, *nuoD* and *trpE* as 5, 17, 98, 12, 14, 4 and 10, respectively, corresponding to the same ST, ST611. A RAPD typing was further carried out on the CSP18 and CRP42 strains and shown in Supplementary Figure 1B. MLST analysis and RAPD typing results indicated that they belonged to the same clone lineage (Diene et al., 2013; Wang et al., 2017; Fraile-Ribot et al., 2018).

**TABLE 1:** MICs (μg/ml) of indicated *P. aeruginosa* strains.

**Strains**	**Ciprofloxacin (μg/ml)^*a*^**	**Levofloxacin (μg/ml)^*a*^**
CSP18	0.25	2
CRP42	8	32
CSP18*gyrA*_CRP__42_	1	4
CRP42*gyrA*_CSP__18_	2	16
CSP18 *mexS*_CRP__42_	2	16
CSP18*gyrA*_CRP__42_*mexS*_CRP__42_	8	32

*^*a*^Clinical Laboratory Standards Institute (CLSI) susceptibility breakpoints: ciprofloxacin ≤ 1 μg/ml, levofloxacin ≤ 2 μg/ml, resistance breakpoints: ciprofloxacin ≥ 4 μg/ml, and levofloxacin ≥ 8 μg/ml.*

### Isolate CRP42 Is Resistant to Ciprofloxacin

Ciprofloxacin is commonly used to treat serious *P. aeruginosa* infections, and ciprofloxacin susceptibility of CSP18 and CRP42 was found different according to the preliminary analysis with VITEK automatic microbe analysis instrument (data not shown). Therefore, we further tested their susceptibility to ciprofloxacin by the twofold serial dilution method (Richardot et al., 2016). As shown in Table 1, initial isolate (CSP18) was found to be susceptible to ciprofloxacin, while the later isolate (CRP42) was resistant to the antibiotic, with a 32-fold increase in MIC of ciprofloxacin. Levofloxacin, another fluoroquinolone, was also examined. Similar to ciprofloxacin, CSP18 was susceptible to levofloxacin, while CRP42 showed resistance to levofloxacin, with a 16-fold increase in MIC of levofloxacin (Table 1). Besides, CRP42 displayed a reduced susceptibility to chloramphenicol, nalidixic acid, norfloxacin, and imipenem, and an increased susceptibility to gentamicin and amikacin, as well as a same susceptibility to tetracycline (Table 2).

**TABLE 2:** MICs (μg/ml) of CSP18 and CRP42 strains.

**Strains**	**Imipenem**	**Chloramphenicol**	**Nalidixic acid**	**Norfloxacin**	**Gentamicin**	**Amikacin**	**Tetracycline**
CSP18	2	32	256	1	4	2	25
CRP42	8	1024	>2048	32	2	1	25

### A D87G Substitution in GyrA Contributes to the Increased Resistance to Ciprofloxacin in CRP42

To elucidate the mechanism of reduced susceptibility to ciprofloxacin in isolate CRP42, genome re-sequencing was carried out to identify mutations in the genome of CRP42 relative to that of CSP18, using PAO1 as reference genome (see text footnote 2). Between strains CSP18 and CRP42, 17 genes had frameshifts (insertion/deletion), while 35 genes had non-synonymous SNV (Supplementary Table 3). Among them, the sequence of *gyrA*, encoding DNA gyrase subunit A, which has been reported to be involved in ciprofloxacin resistance (Higgins et al., 2003), was identical to that of PAO1 in CSP18, while in strain CRP42, an “A” was substituted by “G” at the 260th position, resulting in a D87G substitution in GyrA. To confirm the mutation, *gyrA* genes were amplified and cloned from the genomic DNA of strains CSP18 and CRP42. Sequencing analysis revealed that *gyrA* in CSP18 was the same as PAO1, while gyrA from CRP42 displayed a D87G substitution, which confirmed the genomic re-sequencing results. To test if the D87G substitution in GyrA contributed to the decreased susceptibility of the CRP42 to ciprofloxacin, the *gyrA*_CRP__42_ gene with its native promoter was introduced into the genome of CSP18 via pUC18T-mini-Tn7T plasmid and then the native *gyrA* was knocked out in CSP18 by chromosomal recombination. As shown in Table 1, replacement of the *gyrA*_CSP__18_ with *gyrA*_CRP__42_ resulted in a fourfold increase in MIC against ciprofloxacin in CSP18. Similarly, replacement of the *gyrA*_CRP__42_ with *gyrA*_CSP__18_ decreased the MIC of ciprofloxacin in CRP42 by fourfold. These results indicated that the D87G substitution in GyrA contributed to the reduced susceptibility to ciprofloxacin in strain CRP42 and also suggested the presence of other ciprofloxacin resistance mechanisms in CRP42.

### F7S Mutation in MexS of CRP42 Contributes to the Increased Resistance to Ciprofloxacin

**FIGURE 1:**
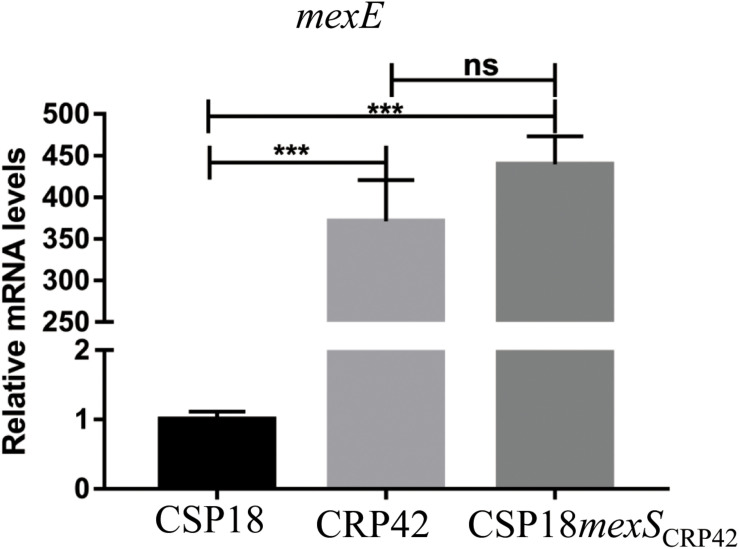
Transcriptional levels of *mexE* gene in indicated strains. Total RNA was extracted from indicated *P. aeruginosa* strains at OD*_600_* of 1.0, and the relative mRNA levels of *mexE* were determined by real-time qPCR with *rpsL* as an internal control. ns, not significant, ***P < 0.001, by Student’s *t* test.

To look for the other possible ciprofloxacin resistance mechanisms, we further compared the global gene expression profiles between strain CSP18 and CRP42. Expression levels of 48 genes were altered between the two strains (Supplementary Table 4). Among them, MexEF-OprN efflux pump encoding genes *mexE*, *mexF*, and *oprN* displayed 239-, 223-, and 169-fold higher mRNA levels, respectively, in CRP42 than those in CSP18 (Supplementary Table 4). To confirm the transcriptional up-regulation, the mRNA levels of *mexEF-oprN* operon were further examined and compared between CSP18 and CRP42 by real-time qPCR. As displayed in Figure 1, consistent with the RNAseq results, the relative mRNA level of *mexE* showed a 370-fold increase in the strain CRP42 compared to that in CSP18. To further elucidate the molecular mechanism of the reduced susceptibility to ciprofloxacin in CRP42, as well as the increased transcriptional levels of the *mexEF-oprN* operon, we further examined the genomic re-sequencing results. Among them, a T20C substitution in the *mexS* gene resulted in an F7S substitution in the MexS, a putative oxidoreductase of the CRP42 strain in comparison to that of CSP18 (Supplementary Table 3). This was further verified by sequencing of the PCR amplicons. To assess if the F7S substitution in MexS contributed to the increased *mexEF-oprN* expression and the decreased susceptibility to ciprofloxacin in CRP42, the chromosomal *mexS* gene was replaced by *mexS*_CRP__42_ in the CSP18 strain. The mRNA levels of *mexE* were compared by real-time qPCR. As the results shown in Figure 1, the replacement by *mexS*_CRP__42_ led to a 439-fold increase in the relative mRNA level of *mexE* in the CSP18 strain, while no significant change of *mexE* mRNA levels was observed with GyrA D87G substitution (Supplementary Figure 2F). To further confirm the increased expression of *mexE* gene, *mexE-lacZ* reporter construct was further introduced into the CSP18*mexS*_CRP__42_ strain. Consistent with the real-time qPCR results, the *mexS* replacement rendered a significant increase in beta-galactosidase activity compared to that of CSP18 strain (Supplementary Figure 2A). Furthermore, as shown in Table 1, replacement of chromosomal MexS with F7S-substituted MexS conferred CSP18 an eightfold increased MIC against ciprofloxacin. These data demonstrated that the F7S substitution in MexS is the cause of the dramatic increase in the expression of *mexEF-oprN* seen in the CRP42 strain. Clearly, the F7S substitution disrupted the repressor function of the MexS. This is a new finding, since F7S point mutation in MexS has not been described to affect the expression of MexEF-OprN up to now.

It has been demonstrated that *mexT*, located downstream of the *mexS*, encodes a LysR-family transcriptional activator for the *mexEF-oprN* (Kohler et al., 1997). We addressed if the MexT is involved in the up-regulation of *mexEF-oprN* and thus the reduced susceptibility to ciprofloxacin in CRP42. The *mexT* genes were PCR amplified from genomes of CSP18 and CRP42 and subjected to sequencing analysis. Consistent with the genomic re-sequencing results, *mexT* in strain CSP18 shares an identical sequence with that of CRP42, with an 8-bp deletion (CGGCCAGC) spanning from nucleotide 225 to 232 (104 to 111) in comparison to the reference strain PAO1 (see text footnote 2; PAO1 lab strain; Jin et al., 2011), while in comparison to PA14, 12 SNVs were observed in nucleotide sequence of *mexT*, resulting in no amino acid change of MexT in both CSP18 and CRP42 strains. Furthermore, no change at the transcriptional level of the *mexT* was observed between CSP18 and CRP42 (Supplementary Figure 2B), which is consistent with previous reports that the impact of *mexS* mutation on *mexEF-oprN* expression was not related to changes in MexT levels (e.g., Sobel et al., 2005; Richardot et al., 2016). These results suggested that MexT was the cause of neither the elevated *mexEF-oprN* transcription nor the increased resistance to ciprofloxacin in CRP42.

Besides MexEF-OprN, MexAB-OprM, MexCD-OprJ and MexXY are involved in ciprofloxacin resistance (Kohler et al., 1997; Masuda et al., 2000; Morita et al., 2012; Rehman et al., 2019). While as shown in Supplementary Figure 2, expression levels of *mexAB-oprM*, *mexCD-oprJ* and *mexXY* are the same between CSP18 and CRP42, and no mutation was found in these genes (data not shown). Thus, they are not responsible for the reduced susceptibility to ciprofloxacin in CRP42.

### Point-Mutated MexS and GyrA Together Are Responsible for the Increased Resistance to Ciprofloxacin in CRP42

We further assessed if the D87G-substituted GyrA, together with the F7S-substituted MexS, was responsible for the reduced susceptibility to ciprofloxacin in strain CRP42. In the CSP18*gyrA*_CRP__42_ background, the *mexS*_CSP18_ gene was further replaced by *mexS*_CRP__42_. As shown in Table 1, replacement of the *mexS*_CSP18_ with *mexS*_CRP__42_ conferred CSP18*gyrA*_CRP__42_ strain (CSP18*gyrA*_CRP__42_*mexS*_CRP__42_) an identical MIC against ciprofloxacin as the CRP42 strain. Replacement of *gyrA*_CSP__18_ with *gyrA*_CRP__42_ in CSP18 (CSP18*gyrA*_CRP__42_) resulted in a fourfold increase in MIC against ciprofloxacin (from 0.25 to 1.0 μg/ml), and the additional replacement of *mexS*_CSP__18_ with *mexS*_CRP__42_ (CSP18*gyrA*_CRP__42_*mexS*_CRP__42_) conferred a further eightfold increase in the MIC (from 1 to 8 μg/ml; Table 1).

### CRP42 Produces Less Pyocyanin and Rhamnolipids Than CSP18

Our RNAseq results also revealed decreased transcriptional levels of *phzM*, *phzA1*-*phzB1*, *phzG1*, and *phzS*, and *rhlA* and *rhlB* (Supplementary Table 4), which are involved in pyocyanin and rhamnolipids production, respectively, (Mavrodi et al., 2001; Zhu and Rock, 2008). Accordingly, we examined and compared the pyocyanin and rhamnolipids production between CSP18 and CRP42 strains. As the results shown in Supplementary Figure 3, the CRP42 strain produced strongly decreased pyocyanin and rhamnolipids than the CSP18 strain. Furthermore, replacement of *mexS* by *mexS*_CRP__42_ decreased the pyocyanin and rhamnolipids production of CSP18 almost to the levels of CRP42 (Supplementary Figure 3), while no obvious change of pyocyanin or rhamnolipids production was observed with *gyrA* substitution (Supplementary Figure 3).

## Discussion

*Pseudomonas aeruginosa* PAO1 genome encodes 12 RND-type efflux pumps (Stover et al., 2000). Among them, MexEF-OprN can exclude ciprofloxacin, chloramphenicol and trimethoprim from bacterial cell (Kohler et al., 1997). Its expression is controlled by multiple factors. *mexT*, located immediately upstream of and transcribed in the same direction as the *mexEF-oprN*, encodes a LysR family transcriptional regulator, capable of activating *mexEF-oprN* operon expression (Kohler et al., 1997). In the strain CSP18, *mexT* shares an identical sequence to that in CRP42, which has an 8-bp deletion (CGGCCAGC) at the 225th position in comparison to that of reference strain PAO1 (see text footnote 2). According to previous reports, in the absence of this 8-bp fragment, MexT is functional (Maseda et al., 2000; Richardot et al., 2016). Therefore, MexT is functional and responsible for the increased expression of *mexEF-oprN* due to the inactivation of MexS in CRP42.

*mexS*, located upstream of and activated by MexT, encodes a putative oxidoreductase/dehydrogenase homology (see text footnote 2). First of all, MexS was found to repress *mexEF-orN* expression independent of the changes in MexT levels in a clinical isolate of *P. aeruginosa* (Sobel et al., 2005). Consistent with that study, F7S substitution in MexS caused a hyper-expression of *mexEF-oprN* in CRP42, with no transcriptional level change in *mexT*. However, the effect of MexS on *mexEF-oprN* is complex. In our previous study with a lab strain PAO1, MexS positively regulated the expression of *mexEF-oprN* (Jin et al., 2011). A more recent study demonstrated that there are two separate pathways regulating the *mexEF-oprN* expression, a MexS-mediated and a MexS-bypassed pathway (Uwate et al., 2013). The complicated regulation of *mexEF-oprN* mediated by MexS may reflect the differences observed in the different *P. aeruginosa* strains. Of note, single point mutation in MexS (A155V/L263Q/S60P/A166P/C245G/F185L/S60F/D44E/F253L), resulting in partially or completely deficient MexS activity as well as *mexEF-oprN* overexpression, has recently been reported in clinical *P. aeruginosa* (Morita et al., 2015; Richardot et al., 2016). Our current study demonstrated that a novel single point mutation, resulting in F7S substitution in MexS, a putative oxidoreductase, caused a significant up-regulation of *mexEF-oprN* in both clinical *P. aeruginosa* strain CRP42 and CSP18*mexS*_CRP__42_. In fact, the F7S substitution was predicted to be deleterious to the MexS’s function using the SIFT algorithm (data not shown)^3^ (Kumar et al., 2009). Consistent with previous reports that mutations in MexS decreased production of outer membrane protein OprD, concomitant with enhanced resistance to imipenem in *P. aeruginosa* (Sobel et al., 2005), CRP42 strain displayed a 3.6-fold decreased mRNA level of *oprD* (data not shown) and a reduced susceptibility to imipenem compared to CSP18 (Table 2).

Expression of *mexEF-oprN* is also regulated by MvaT, a global regulator of *P. aeruginosa* virulence genes. MvaT negatively regulates *mexEF-oprN* expression independent of *mexT* and *mexS* (Westfall et al., 2006). Besides, CmrA, an AraC-like transcriptional regulator, negatively controls *mexEF-oprN* expression through *mexS* and *mexT* (Juarez et al., 2017). MxtR, a sensor kinase, has recently been reported to repress *mexEF-oprN* via MexT in *P. aeruginosa* (Zaoui et al., 2012). In the present study, none of the MvaT, CmrA or MxtR encoding genes displayed different nucleotide sequences between CSP18 and CRP42 (data not shown), thus unlikely the cause of increased *mexEF-oprN* expression in CRP42.

It has been reported that MexEF-OprN can also export signal molecules of quorum sensing, and hyper-production of MexEF-OprN reduced production of several quorum sensing-dependent extracellular virulence factors, such as pyocyanin and rhamnolipids (Kohler et al., 2001). Consistent with the previous study, transcriptional levels of genes related to phenazine biosynthesis displayed 6.8- to 30-fold decreases in CRP42 (Supplementary Table 4), and production of pyocyanin in CRP42 was significantly decreased than that in CSP18 (Supplementary Figure 3). Similarly, the CRP42 strain displayed a 10-fold decreased transcriptional level in *rhlA* and *rhlB*, two rhamnolipids-related genes (Supplementary Table 4), and a strongly decreased production of rhamnolipids. Replacement by *mexS*_CRP__42_ conferred CSP18 strain a CRP42-like expression level of *mexEF-oprN* (Figure 1), as well as production of pyocyanin and rhamnolipids (Supplementary Figure 3).

Previous studies have shown that T83I substitution in GyrA is the most common alteration associated with ciprofloxacin resistance in *P. aeruginosa* clinical isolates (Higgins et al., 2003; Lee et al., 2005; Pasca et al., 2012; Bruchmann et al., 2013). Substitutions of aspartate at position 87 with asparagine, tyrosine, or glycine residues have also been reported among ciprofloxacin-resistant clinical *P. aeruginosa* isolates (Higgins et al., 2003; Lee et al., 2005; Pasca et al., 2012; Bruchmann et al., 2013). Although the possible contribution of the D87G substitution in GyrA to ciprofloxacin resistance had been suggested, the quantitative contribution of the D87G substitution in ciprofloxacin resistance has not been examined for *P. aeruginosa*. Our study demonstrated that D87G mutation in GyrA renders a fourfold increase in MIC of ciprofloxacin.

The previous study used the well-known RND-type multidrug efflux pump inhibitor Phe-Arg-β-naphthylamide (PAβN) to study relative contribution of efflux pump up-regulation and target gene mutations to ciprofloxacin resistance in clinical *P. aeruginosa* and found that it was complex (Dunham et al., 2010). In the present study, we simulated the point mutation of genes *gyrA* and *mexS* in the ciprofloxacin-susceptible strain CSP18 by gene editing to its ciprofloxacin-resistant derivative and demonstrated the detailed quantitative contribution of the D87G point mutation of target gene *gyrA* and MexEF-OprN hyper-expression caused by F7S point mutation in MexS.

## Data Availability Statement

The datasets presented in this study can be found in online repositories. The names of the repository/repositories and accession number(s) can be found in the article/Supplementary Material.

## Author Contributions

YJ conceived and designed the experiments. CX, HL, XP, ZM, DW, XZ, and GZ performed the experiments. YJ, FB, ZC, and WW analyzed the data. YJ wrote the manuscript. All authors contributed to the article and approved the submitted version.

## Conflict of Interest

The authors declare that the research was conducted in the absence of any commercial or financial relationships that could be construed as a potential conflict of interest.

## Footnotes

1 http://pubmlst.org/paeruginosa/

2 www.pseudomonas.com

3 http://sift.jcvi.org

## Supplementary Material

Supplementary Figure 1 PCR results of indicated strains. **(A)** 16S rDNA gene amplification; **(B)** RAPD typing of indicated strains.

Supplementary Figure 2 Transcriptional levels of indicated genes in indicated strains. **(A)** β-galactosidase assay was conducted to determine the transcriptional activity of *mexE* gene promoter fused to a *lacZ* gene in indicated strains. **(B–E)** Relative mRNA levels of *mexT* **(B)**, *mexB* **(C)**, *mexC* **(D)**, *mexY* **(E)**, and *mexE* **(F)** in indicated strains; Total RNA was extracted from indicated *P. aeruginosa* strains at OD*_600_* of 1.0, and the relative mRNA levels of indicated genes were determined by real-time qPCR with *rpsL* as an internal control. ns, not significant, ^∗∗∗^*P* < 0.001, by student’s *t* test.

Supplementary Figure 3 Pyocyanin and rhamnolipids production by indicated strains. Overnight culture of indicated strains **(A)** were diluted 50-fold and grown for 24 h. **(B)** 1 mL supernatant of each bacterial culture was extracted into 0.5 mL of chloroform, then 0.4 mL of which was re-extracted into 0.3 mL of HCl (0.2N), and subjected to measurement at OD*_520_*. ns, not significant, ^∗∗^*P* < 0.01 by Student’s *t* test. **(C)** Rhamnolipids production were determined on the plate. 1 microliter culture of the indicated *P. aeruginosa* strain was inoculated onto the plate, grown at 37°C for 24 h and then at room temperature for another 72 h. The presence of a halo around the colonies suggests the production of rhamnolipids. The diameter ratio means the diameter of each halo divided by the diameter of its colony. The data shown represent means ± standard errors.

Supplementary Table 1 Strains and plasmids used in this work.

Supplementary Table 2 Primers used in the work.

Supplementary Table 3 SNVs identified by genomic re-sequencing with elimination of SNVs with synonymous mutation and the same mutations between CSP18 and CRP42.

Supplementary Table 4 Transcriptome analysis: differentially expressed genes by RNAseq.
