# Supplementary figures and images for: Mechanisms for Development of Ciprofloxacin Resistance in a Clinical Isolate of Pseudomonas aeruginosa
Source: Front Microbiol. 2021 Jan 8;11:598291. doi: 10.3389/fmicb.2020.598291 (PMC7819972; doi:10.3389/fmicb.2020.598291)

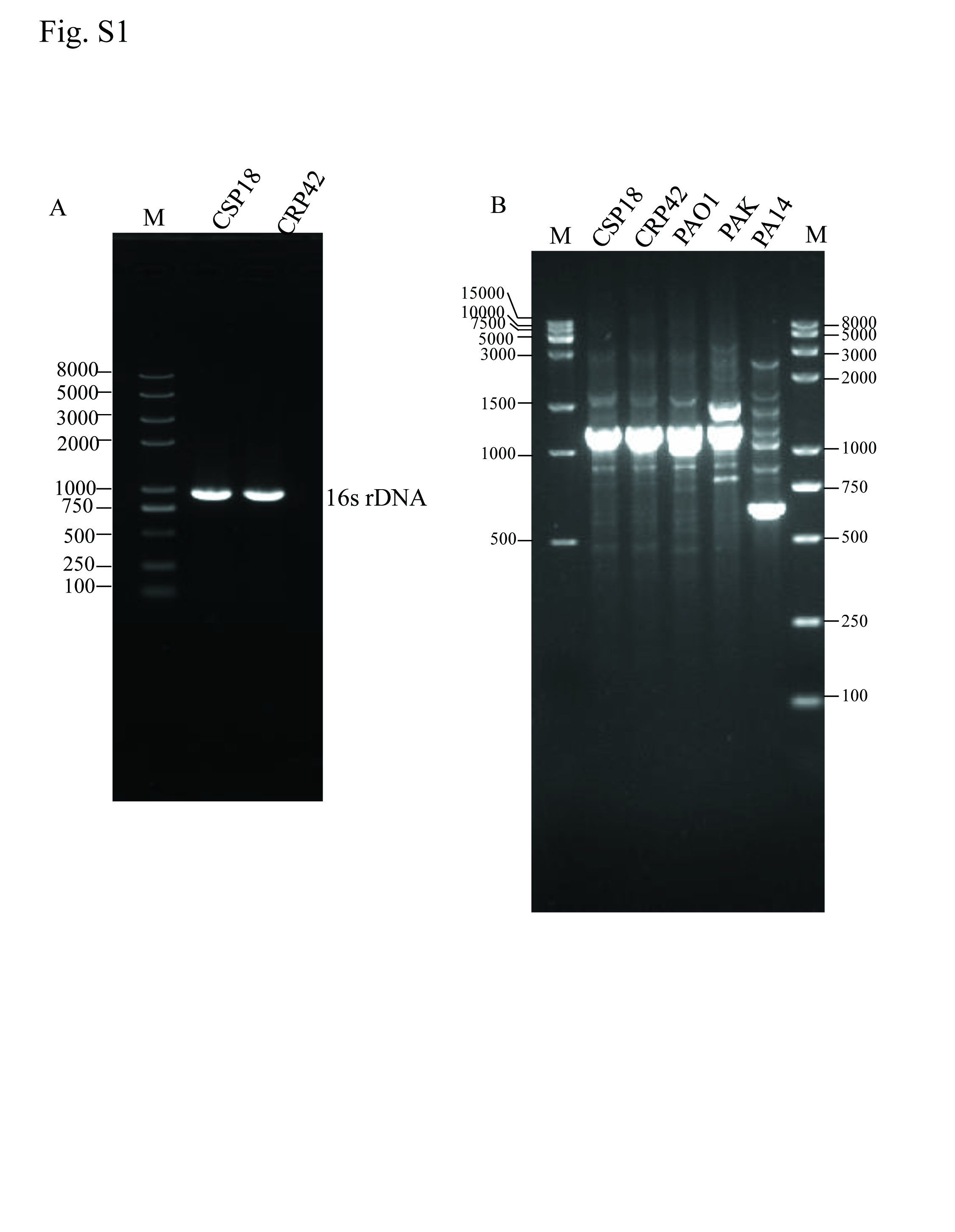

Supplement: Supplementary Figure 1 — PCR results of indicated strains. (A) 16S rDNA gene amplification; (B) RAPD typing of indicated strains. [file Image_1.TIF]

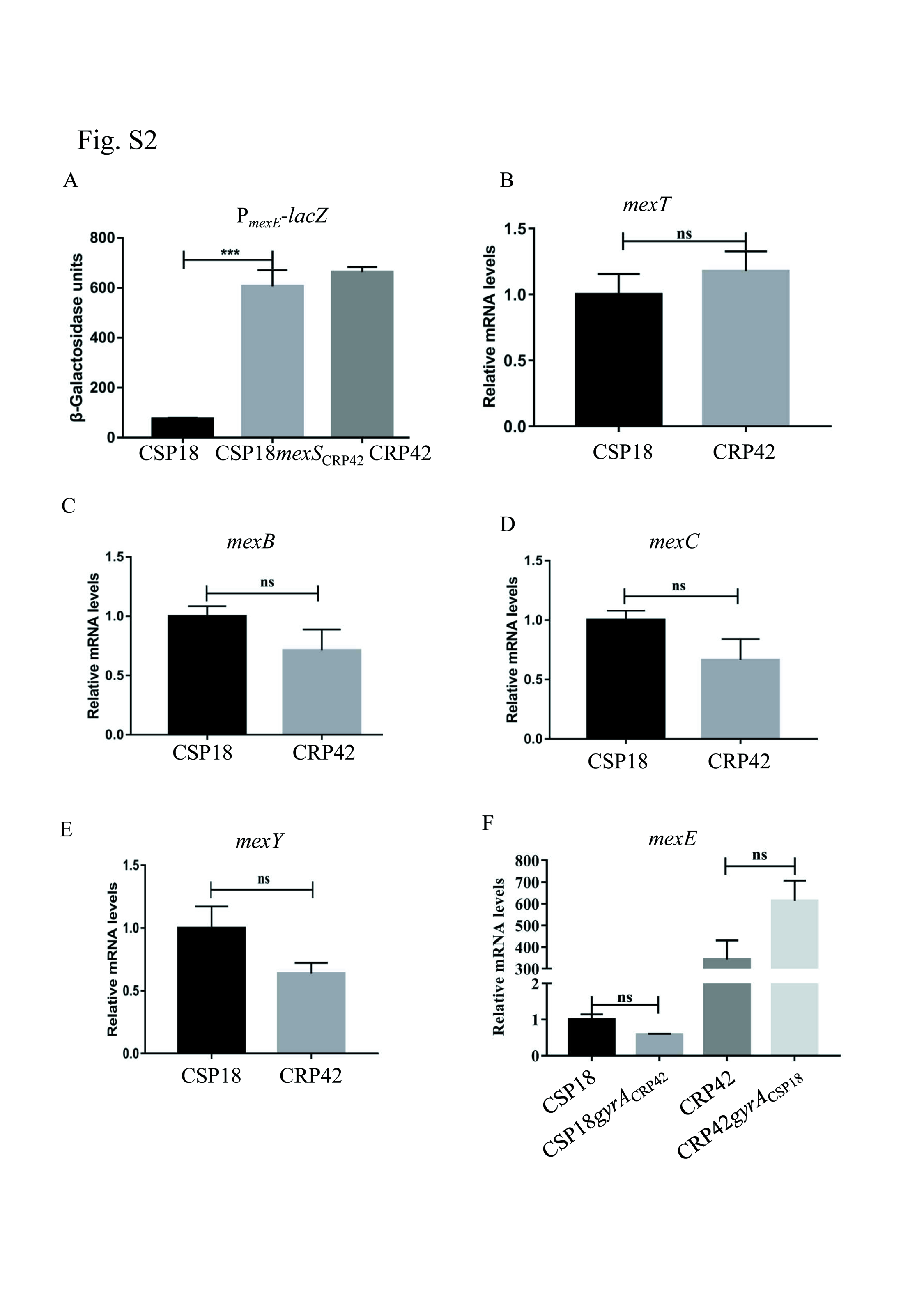

Supplement: Supplementary Figure 2 — Transcriptional levels of indicated genes in indicated strains. (A) β-galactosidase assay was conducted to determine the transcriptional activity of mexE gene promoter fused to a lacZ gene in indicated strains. (B–E) Relative mRNA levels of mexT (B), mexB (C), mexC (D), mexY (E), and mexE (F) in indicated strains; Total RNA was extracted from indicated P. aeruginosa strains at OD600 of 1.0, and the relative mRNA levels of indicated genes were determined by real-time qPCR with rpsL as an internal control. ns, not significant, ∗∗∗P < 0.001, by student’s t test. [file Image_2.tif]

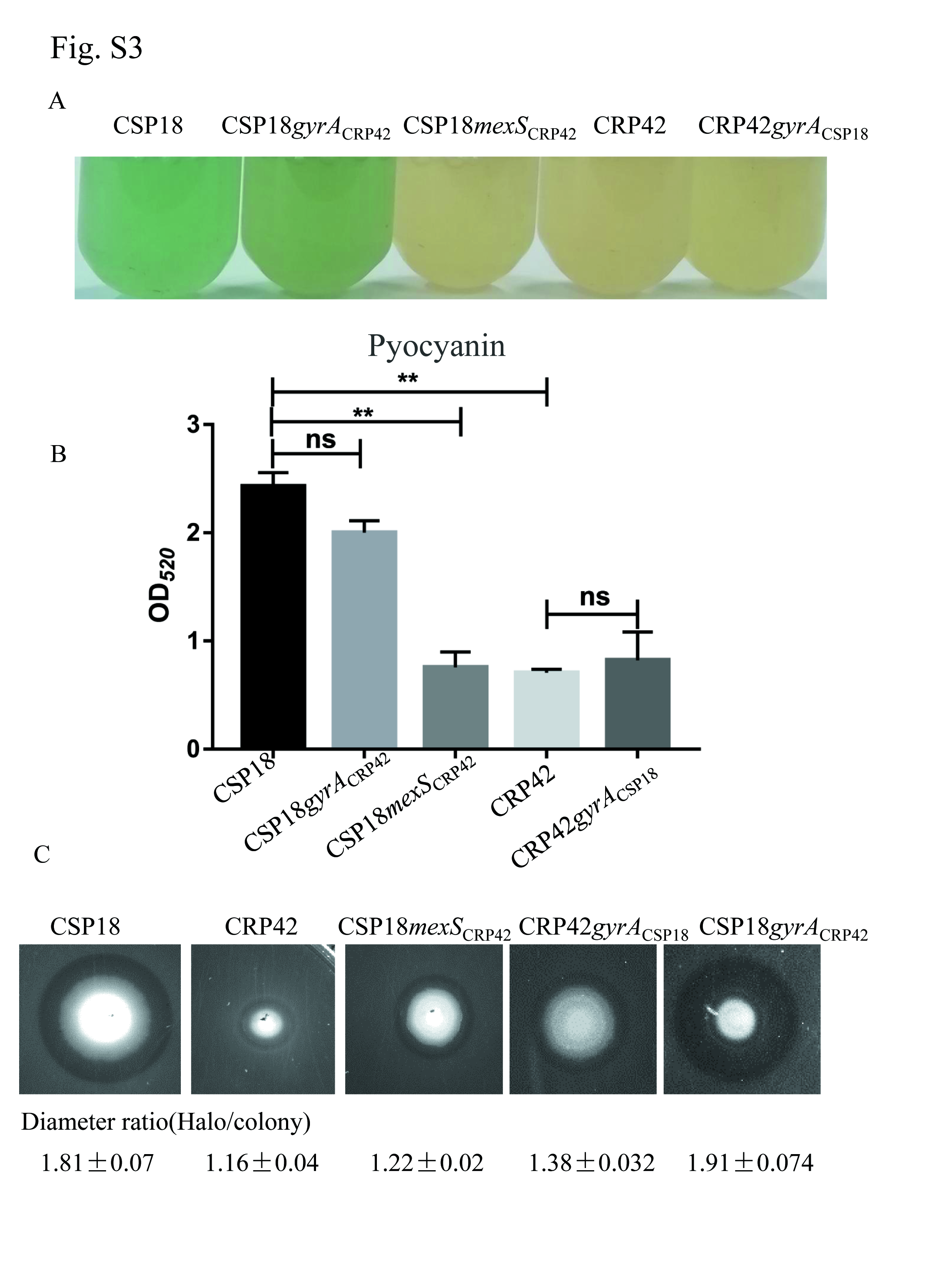

Supplement: Supplementary Figure 3 — Pyocyanin and rhamnolipids production by indicated strains. Overnight culture of indicated strains (A) were diluted 50-fold and grown for 24 h. (B) 1 mL supernatant of each bacterial culture was extracted into 0.5 mL of chloroform, then 0.4 mL of which was re-extracted into 0.3 mL of HCl (0.2N), and subjected to measurement at OD520. ns, not significant, ∗∗P < 0.01 by Student’s t test. (C) Rhamnolipids production were determined on the plate. 1 microliter culture of the indicated P. aeruginosa strain was inoculated onto the plate, grown at 37°C for 24 h and then at room temperature for another 72 h. The presence of a halo around the colonies suggests the production of rhamnolipids. The diameter ratio means the diameter of each halo divided by the diameter of its colony. The data shown represent means ± standard errors. [file Image_3.TIF]
