# Supplementary material for: Mechanisms for Development of Ciprofloxacin Resistance in a Clinical Isolate of Pseudomonas aeruginosa
Source: Front Microbiol. 2021 Jan 8;11:598291. doi: 10.3389/fmicb.2020.598291 (PMC7819972; doi:10.3389/fmicb.2020.598291)
Supplement: Supplementary Table 1 — Strains and plasmids used in this work. [file Table_1.DOCX]

**Table S1. Strains and plasmids used in this work**

| **Strains/plasmids** | **Description** | **Source/reference**  **(reference)** |
| --- | --- | --- |
| **Strains** |  |  |
| CSP18 | A ciprofloxacin sensitive clinical isolate | This study |
| CRP42 | A ciprofloxacin resistant clinical isolate | This study |
| CSP18*gyrA*_CRP42_ | CSP18 with its *gyrA* replaced by that of CRP42 | This study |
| CSP18*mexS*_CRP42_ | CSP18 with its *mexS* replaced by that of CRP42 | This study |
| CRP42*gyrA*_CSP18_ | CRP42 with its *gyrA* replaced by that of CSP18 | This study |
| CSP18*gyrA*_CRP42_*mexS*_CRP42_ | CSP18 with its *gyrA and mexS* replaced by those of CRP42 | This study |
| **Plasmids** |  |  |
| pUC18T-mini-Tn7T-Gm | Mini-Tn7 base vector for insertion into chromosome attTn7 site; Gm^r^ | ([Choi and Schweizer, 2006](#_ENREF_1)) |
| pUC18T-*gyrA* _CSP18_ | *gyrA* from CSP18 cloned into pUC18T-mini-Tn7T-Gm | This study |
| pUC18T-*gyrA* _CRP42_ | *gyrA* from CRP42 cloned into pUC18T-mini-Tn7T-Gm | This study |
| pEX18Tc | Gene replacement vector; Tc^r^, *oriT*^+^, *sacB*^+^ | ([Hoang et al., 1998](#_ENREF_2)) |
| pEX18-*gyrA* | *gyrA* gene deletion construct in pEX18Tc; Tc^r^ | This study |
| pEX18-*mexS* | *mexS* F7S point mutation construct in pEX18Tc; Tc^r^ | This study |
| pTNS3 | Helper plasmid, for gene insertion into chromosome; Amp^r^ | ([Choi and Schweizer, 2006](#_ENREF_1)) |
| P*_mexE_*-*lacZ* | *mexE* promoter fused to *lacZ* | ([Jin et al., 2011](#_ENREF_3)) |

Choi, K.H., and Schweizer, H.P. (2006). mini-Tn7 insertion in bacteria with single attTn7 sites: example *Pseudomonas aeruginosa*. *Nat Protoc* 1(1)**,** 153-161. doi: 10.1038/nprot.2006.24.

Hoang, T.T., Karkhoff-Schweizer, R.R., Kutchma, A.J., and Schweizer, H.P. (1998). A broad-host-range Flp-FRT recombination system for site-specific excision of chromosomally-located DNA sequences: application for isolation of unmarked *Pseudomonas aeruginosa* mutants. *Gene* 212(1)**,** 77-86. doi: 10.1016/s0378-1119(98)00130-9.

Jin, Y., Yang, H., Qiao, M., and Jin, S. (2011). MexT regulates the type III secretion system through MexS and PtrC in *Pseudomonas aeruginosa*. *J Bacteriol* 193(2)**,** 399-410. doi: 10.1128/jb.01079-10.

West, S.E., Schweizer, H.P., Dall, C., Sample, A.K., and Runyen-Janecky, L.J. (1994). Construction of improved *Escherichia-Pseudomonas* shuttle vectors derived from pUC18/19 and sequence of the region required for their replication in *Pseudomonas aeruginosa*. *Gene* 148(1)**,** 81-86. doi: 10.1016/0378-1119(94)90237-2.
