# Supplementary material for: Mechanisms for Development of Ciprofloxacin Resistance in a Clinical Isolate of Pseudomonas aeruginosa
Source: Front Microbiol. 2021 Jan 8;11:598291. doi: 10.3389/fmicb.2020.598291 (PMC7819972; doi:10.3389/fmicb.2020.598291)
Supplement: Supplementary Table 2 — Primers used in the work. [file Table_2.DOCX]

**Table S2. Primers used in the work**

| **Primers** ^a^ | **Sequence (5’→3’)** | **Use** | **Reference/Source** |
| --- | --- | --- | --- |
| *acsA*-F | ACCTGGTGTACGCCTCGCTGAC | Gene amplification for MLST analysis | Curran *et al* (2004) |
| *acsA*-R | GACATAGATGCCCTGCCCCTTGAT |  |  |
| *aroE*-F | TGGGGCTATGACTGGAAACC |  |  |
| *aroE*-R | TAACCCGGTTTTGTGATTCCTACA |  |  |
| *guaA*-F | CGGCCTCGACGTGTGGATGA |  |  |
| *guaA*-R | GAACGCCTGGCTGGTCTTGTGGTA |  |  |
| *mutL*-F | CCAGATCGCCGCCGGTGAGGTG |  |  |
| *mutL*-R | CAGGGTGCCATAGAGGAAGTC |  |  |
| *nuoD*-F | ACCGCCACCCGTACTG |  |  |
| *nuoD*-R | TCTCGCCCATCTTGACCA |  |  |
| *ppsA*-F | GGTCGCTCGGTCAAGGTAGTGG |  |  |
| *ppsA*-R | GGGTTCTCTTCTTCCGGCTCGTAG |  |  |
| *trpE*-F | GCGGCCCAGGGTCGTGAG |  |  |
| *trpE*-R | CCCGGCGCTTGTTGATGGTT |  |  |
| 16s rDNAF | GGGGGATCTTCGGACCTCA | 16s rDNA PCR/sequencing | Spilker *et al* (2004) |
| 16s rDNAR | TCCTTAGAGTGCCCACCCG |  |  |
| RAPD primer | AGGAAGGTGC | RAPD analysis  Gene sequencing for MLST analysis | Mahenthiralingam et al (1996) |
| *acsA*-SF | GCCACACCTACATCGTCTAT | Gene sequencing for MLST analysis | Curran *et al* (2004) |
| *acsA*-SR | AGGTTGCCGAGGTTGTCCAC |  |  |
| *aroE*-SF | ATGTCACCGTGCCGTTCAAG |  |  |
| *aroE*-SR | TGAAGGCAGTCGGTTCCTTG |  |  |
| *guaA*-SF | AGGTCGGTTCCTCCAAGGTC |  |  |
| *guaA*-SR | GACGTTGTGGTGCGACTTGA |  |  |
| *mutL*-SF | AGAAGACCGAGTTCGACCAT |  |  |
| *mutL*-SR | GGTGCCATAGAGGAAGTCAT |  |  |
| *nuoD*-SF | ACGGCGAGAACGAGGACTAC |  |  |
| *nuoD*-SR | TGGCGGTCGGTGAAGGTGAA |  |  |
| *ppsA*-SF | GGTGACGACGGCAAGCTGTA |  |  |
| *ppsA*-SR | GTATCGCCTTCGGCACAGGA |  |  |
| *trpE*-SF | TTCAACTTCGGCGACTTCCA |  |  |
| *trpE*-SR | GGTGTCCATGTTGCCGTTCC |  |  |
| *mexS*F | CGGGATCCACGACGGGTGGTTTTGTCAGGC | *mexS* amplification | This study |
| *mexS*R | CCCAAGCTTGGACGGCGGTCAACGATCTGTG |  |  |
| *mexT*F | CGGGATCCGATCACTCGGGACATCGCAAAC | *mexT* amplification | This study |
| *mexT*R | CCCAAGCTTATGGAATAAGCCGCACACCCG |  |  |
| *gyrA*F | CCCAAGCTTTACTTCGTTTGCCTCAGGATCG | *gyrA* amplification | This study |
| *gyrA*R | CCGAGCTCTCGCTTGCTCACATCCACTCTC |  |  |
| q*mexE*F | ATCAAGGACGAAGCGGTC | RT-qPCR  RT-qPCR | This study |
| q*mexE*R | GTAGACGGTCTTGTTGTC |  |  |
| q*mexT*F | GATCTGAACCTGCTGATC | RT-qPCR  RT-qPCR | This study |
| q*mexT*R | CGAGGAACAGTTTCTCTG |  |  |
| q*mexB*F | GGTGAAGAACTTCCTCAT | RT-qPCR  RT-qPCR | This study |
| q*mexB*R | TGTTGGAAACGATGTAGT |  |  |
| q*mexC*F | GTACCGGCGTCATGCAGGGTTC | RT-qPCR  RT-qPCR | This study |
| q*mexC*R | TTACTGTTGCGGCGCAGGTGACT |  |  |
| q*mexY*F | CTCGGTGTTGATCGTGTTCC | RT-qPCR  RT-qPCR | This study |
| q*mexY*R | GATGAGGATGGCGTTCTTCG |  |  |
| *gyrA*UF | CGGAATTCACCTGACCATGGCCCAGTTG | *gyrA* deletion | This study |
| *gyrA*UR | GGGGTACCACCGGGAGAATTTCTTTGGC |  |  |
| *gyrA*DF | GG GGTACGCGGAAGCGGAAGGCAACGAAG |  |  |
| *gyrA*DR | CGGGATCCGCCGGGGTGTTGTACATGGAAC |  |  |
| *mexS*UF | CGGAATTCCCCCGAGGCCAGCAGGTTCGGCATC | *mexS* point mutation | This study |
| *mexS*UR | CGGGCCAAACTGATGAGATCTGATCACTCGGG |  |  |
| *mexS*DF | CCCGAGTGATCAGATCTCATCAGTTTGGCCCG |  |  |
| *mexS*DR | CCAAGCTTCTCGTCGTTGCGTTCCAGGCCCATC |  |  |
| *gyrA*F | CCCAAGCTTATCGACATGAACCTGGAGAG | clone *gyrA* into pUC18T-mini-Tn7T | This study |
| *gyrA*R | CCGAGCTCGCTTGCTCACATCCACTCTC |  |  |
| *mexS*TF | AGGGGCTAGAGGCTGCAACCGC | test *mexS* point mutation | This study |
| *mexS*TR | CATAGGCCGGATAATGATCGGG |  |  |

a: F, forward; R, reverse; S, sequencing; U, upstream; D, downstream; q, RT-qPCr; T, test.

Curran, B., Jonas, D., Grundmann, H., Pitt, T., and Dowson, C.G. (2004). Development of a multilocus sequence typing scheme for the opportunistic pathogen *Pseudomonas aeruginosa*. *J Clin Microbiol* 42(12)**,** 5644-5649. doi: 10.1128/jcm.42.12.5644-5649.2004.

Mahenthiralingam, E., Campbell, M.E., Foster, J., Lam, J.S., and Speert, D.P. (1996). Random amplified polymorphic DNA typing of *Pseudomonas aeruginosa* isolates recovered from patients with cystic fibrosis. *J Clin Microbiol* 34(5)**,** 1129-1135.

Spilker, T., Coenye, T., Vandamme, P., and LiPuma, J.J. (2004). PCR-based assay for differentiation of *Pseudomonas aeruginosa* from other *Pseudomonas* species recovered from cystic fibrosis patients. *J Clin Microbiol* 42(5)**,** 2074-2079.
